# Consumer food stockpiling behavior and willingness to pay for food reserves in COVID-19

**DOI:** 10.1007/s12571-020-01092-1

**Published:** 2020-08-06

**Authors:** Erpeng Wang, Ning An, Zhifeng Gao, Emmanuel Kiprop, Xianhui Geng

**Affiliations:** 1 School of Economics and Management, Nanjing Tech University, Nanjing, 211816 China; 2 College of Economics and Management, Nanjing Agricultural University, Nanjing, 210095 China; 3 Food and Resource Economics Department, University of Florida, Gainesville, FL 32611-0240 USA

**Keywords:** Food stockpiling behavior, Food reserves scale, WTP, COVID-19

## Abstract

Consumer behavior changes differently in emergencies. Understanding consumer food stockpiling behavior during COVID-19 pandemic can provide critical information for governments and policymakers to adjust inventory and response strategies. This paper analyzed consumer food stockpiling behavior, including the change of food reserve scale and willingness to pay for fresh food reserves in COVID-19. Our paper shows that the scale of food reserve extends from 3.37 to 7.37 days after the outbreak of COVID-19; if available, consumers on average are willing to pay 18.14 yuan (60.47%) premium for fresh products reserves. The result shows that food stockpiling behavior is fueled by a set of multiple motivations and subjective risk perception. Female, high education level and high-income consumers were more likely to reserve larger scale food reserves, but consumers’ willingness to pay for fresh food reserves is determined by income. This study was conducted when new infection cases continued to rise in the world. The story of food stockpiling during the COVID-19 pandemic in China is similar with the rest of world. Consumer stockpiling behavior in China can also be expanded to other countries to predict the change of food demand and understand more about consumer preferences in emergencies.

## Introduction

Understanding consumer food stockpiling behavior in COVID-19 pandemic can provide critical information for governments and policymakers to adjust inventory and response strategies. COVID-19 is a disease caused by a virus strain that began spreading among people in December 2019. Recently, the continued rising spread of COVID-19 in the world threatens food supply chain and inspires consumer concern about food security. In order to ensure family food security, many consumers rushed to stockpile food, especially fresh agricultural products, which is similar with the situation when Hurricane Sandy struck New York City in 2012 (Bloomberg 2012). Consumers’ food stockpiling behavior results in some empty store shelves, which poses a serious impact on the food system. In this situation, more information about consumer food stockpiling behavior in COVID-19 should be provided to governments and policymakers to understand and respond to the consumer food stockpiling behavior during emergencies.

Consumer stockpiling behavior has been frequently observed, where many consumers buy unusually large amounts of products to avoid possible future shortage or rising prices (Su 2010; Shou et al. 2013). For example, when Hurricane Katrina disabled most oil drilling facilities in the US Gulf coast region in 2005, consumer stockpiling behavior and long lines were observed at gasoline stations. Amid the earthquake and nuclear crisis in Japan in 2011, worried consumers stockpiled salt in Beijing, Shanghai, Chongqing, and other cities (China Daily 2011). The coronavirus pandemic has a strong shock on the food market, which encouraged many families to build or expand their food reserves in order to ensure regular levels of consumption. These panic-bought items may far exceed normal consumption levels and will eventually be wasted. The panic buying and stockpiling phenomenon is a complex and pernicious consumer behavior, fueled by a set of multiple motivations and psychological processes (Dholakia 2020).

Although there exist numerous previous studies of consumer behavior in normal circumstances (Wang et al. 2018; Gao et al. 2019), no such studies exist about consumer stockpiling behavior and preferences in emergencies. Consumer behavior and preference are quite special and different from the common scenario (Kurihara et al. 2012). Increasing consumer food stockpiling behavior during the COVID-19 pandemic raises the following questions to be analyzed: how many days’ food reserves would consumers stockpile during COVID-19? If available, how much are consumers willing to pay for fresh food reserves under the risk of COVID-19?

With use of real purchase data and payment card survey data, the purpose of this paper is to analyze consumers’ food stockpiling behavior during COVID-19 pandemic, identify the change and motivation of food stockpiling behavior, analyze consumers’ willingness to pay for fresh food reserves, and identify the impact factors. This study is the first of this kind to understand consumer food stockpiling behavior and preferences in emergent situations, and can provide useful information for society to cope with COVID-19. Recently, while the spread of the COVID-19 in China has been effectively controlled, new infection cases continue rising in the rest of world. Obvious changes of consumers’ behaviors in light of COVID-19 pandemic have been observed in China. This study of consumer panic buying and stockpiling behavior in China can help us predict the change of food consumption and understand more about consumer behavior in an emergency.

## Literature review

An individual’s preference and behavior are likely to change with disasters and accidents (Teng et al. 2015; Rajesh 2018; Cogato et al. 2019), as revealed in the literature on the endogenous formation of individual preferences that these preferences are not constant across time and change under some circumstances (Fehr and Hoff 2011; Chuang and Schechter 2015; Yusuke and Yasuyuki 2019). In this situation, stockpiling the scarce food will become very common.

The food stockpiling behavior is a complex consumer behavior, driven by a series of multiple motivations and psychological processes (Dholakia 2020). The psychology behind consumer stockpiling indicates that panic buying can be understood as playing to our three fundamental psychological needs; it is about “taking back control” in a world where you feel out of control. Studies summarized by Slovic (1987) identify three clusters of attributes that describe how people perceive various technological hazards and risky activities. These factors may also influence WTP to reduce risk (McDaniels et al. 1992; Savage 1993). One cluster of attributes concerns the extent to which a given risk is source of “dread.” Dreaded risks are perceived as uncontrolled, fatal, and having catastrophic potential. A second cluster involves attributes that are perceived as “unknown,” including risks that are new, unobservable, and unfamiliar and have delayed consequences. A third cluster of attributes concerns an individual’s level of exposure to the risk, and encompasses both personal and societal levels exposure. Risks that are perceived to be unknown and uncontrollable tend to elicit greater fear (Slovic 1987). Our results are largely consistent with Callen et al. (2014) and Hanaoka et al. (2018), supporting the emotional channel behind the nexus between disasters and preferences. This study aims to close the aforementioned gap in the literature on the nexus between disaster and preferences.

## Methodology

The survey was divided into two sections. The first part included questions about consumer real food stockpiling behavior and risk perception in COVID-19. We compare the change of food stockpiling behavior before and after the spread of the novel coronavirus. Real food stockpiling behaviors were reported by respondents, by answering the following questions: How many days’ food reserves did you used to stockpile before COVID-19? How many days’ food reserves do you stockpile during COVID-19? The scale of food reserves includes 1 day, 3 days, 5 days, 1 week, 2weeks, 3 weeks, and 1 month.

In the second section, we use contingent valuation (CV), a popular stated preference method (Hanemann 1984; Hoyos and Mariel 2010) to study consumer preferences of fresh food reserves in COVID-19. Assume there is a package of fresh food reserves that includes a combination of vegetables, fruits, and meats, which can satisfy a three-member family 1-day consumption. We firstly ask respondents to assume that the price for a package of fresh food reserves is 30 yuan before the outbreak of COVID-19, then requested the respondents to pick the maximum acceptable price for the same package of fresh food reserves during COVID-19. In this study, we hire the payment card (PC) approach which is prevalent in the current literature (Hackl and Pruckner 1999; Yu et al. 2014; Wang and Gao 2017). Hence, we set the choices of prices in the payment card to include 7 intervals: 30–40 yuan, 40–50 yuan, 50–60 yuan, 60–70 yuan, 70–80 yuan, 80–90 yuan, 90 yuan or above.

An ordered logit model was used to study what affects consumers’ food reserve scale. We model that consumers stockpile food in order to ensure their family food security, subject to a budget constraint. The scale of food reserve is a function of motivations, risk perception, and demographic characteristics (age, gender, income, and education).

$$ {y}_i^{\ast }={\beta}_1{\mathrm{motivation}}_i+{\beta}_1\mathrm{risk}\_{\mathrm{perception}}_i+{\beta}_1{\mathrm{demo}}_i+{\varepsilon}_i $$

where motivation_i_ represents what motivates respondents to stockpile food, risk _ perception_i_ includes respondents’ perceived infectiousness and perceived own risk for infection of COVID-19. demo_i_ includes demographic characteristics.${\mathrm{y}}_{\mathrm{i}}^{\ast }$ represents the utility that consumer derived from different scales of food reserves, but it is unobserved. We do observe that

$$ {y}_i=1\kern0.75em if\kern0.75em {y}_i^{\ast}\le {\mu}_0 $$

$$ {y}_i=3\kern0.75em if\kern0.5em {\mu}_0<{y}_i^{\ast}\le {\mu}_1 $$

$$ {y}_i=5\kern0.75em if\kern0.5em {\mu}_1<{y}_i^{\ast}\le {\mu}_2 $$

$$ {y}_i=7\kern0.5em if\kern0.5em {\mu}_2<{y}_i^{\ast}\le {\mu}_3 $$

$$ {y}_i=14\kern0.75em if\kern0.5em {\mu}_3<{y}_i^{\ast}\le {\mu}_4 $$

$$ {y}_i=21\kern0.75em if\kern0.5em {\mu}_4<{y}_i^{\ast}\le {\mu}_5 $$

$$ {y}_i=30\kern0.75em if\kern0.5em {\mu}_5<{y}_i^{\ast } $$

The *μ*_*s*_ are unknown threshold parameters to be estimated with *βs*. The probability that consumer *i* will select a specific response j is given by

$$ Prob\ \left({y}_i=j\right)=F\left({\mu}_j-{X}_i^{\prime}\beta \right)-F\left({\mu}_{j-1}-{X}_i^{\prime}\beta \right) $$

where *F* is a cumulative standard logistic distribution.

Furthermore, an interval regression was used to estimate what affects consumers’ willingness to pay for fresh food reserves. The payment card method was used to measure consumers’ willingness to pay for a package of fresh food reserves, so the WTP from our survey consists of intervals and censoring observation. The interval regression is the main technique to tackle such a data structure (Yang et al. 2012), and has been frequently used recently (Yu et al. 2014; Wang and Gao 2017).

## Data collection and description

Online survey and a payment card approach were used to explore consumer food stockpiling behavior. An online survey was conducted by a professional marketing research company (SO JUMP), which is from 11 to 13 March (42% at 11th, 30% at 12th, 28% at 13th) after the outbreak of COVID-19. All samples were randomly selected from 2.6 million sample database and its system can control one IP, one computer, and one account for only one questionnaire. The “trap question” method (Gao et al. 2012, 2016a; Jones et al. 2015) was also used to identify the respondents who may not have carefully read the survey questions. Removing respondents who fail the “trap questions” and those with any missing responses, 1188 valid samples have been collected for this survey. The data includes samples from Beijing, Shanghai, Hubei, Guangdong, Zhejiang, Jiangsu, Hebei, and Shanxi. Hubei is the coronavirus epicenter of the China, which is the most seriously affected. Guangdong and Zhejiang are the second most infectious regions after Hubei; Jiangsu, Hebei, and Shaanxi represent the areas with the less risk of epidemic infection. Beijing is the political and cultural center of China; Shanghai is representative of a global financial center.

Table 1 gives the explanations for the variables and shows the descriptive statistics. The variables include the scale of food reserve before and after the outbreak of COVID-19, WTP for fresh food reserves, whether living in cities, and the demographic characteristics, such as gender, age, education level, and personal monthly income. About 44.7% of the survey respondents were female. 34.3% of the respondents aged more than 35 years old. 54.4% of respondents live in big cities. When they were asked “how long do you think the epidemic still will last from now on,” the mean of respondents’ perceived duration of the COVID-19 is 2.15 months.

**Table 1:** Descriptive statistics

Variable	Description	Whole sample	
		Mean	Std. Dev
WTP	Willingness to pay for fresh products reserve	18.148	13.799
The scale of food reserve before COVID-19	The scale of food reserve before COVID-19	3.367	2.822
The scale of food reserve during COVID-19	The scale of food reserve during COVID-19	7.374	5.695
Female	Female = 1; male = 0	0.447	0.497
Age	(Respondents’ age > 35) = 1;otherwise = 0	0.343	0.413
Education level	More than 12 years education = 1; otherwise = 0	0.782	0.475
Income1	(Monthly personal income < 4000) = 1; otherwise = 0	0.290	0.454
Income2	(4000 ≤ Monthly personal income < 8000) = 1; otherwise = 0	0.371	0.483
Income3	(8000 ≤ Monthly personal income) = 1; otherwise = 0	0.339	0.474
Duration	Perceived duration of the COVID-19 (month)	2.150	1.326
Location	Living in a big city = 1; otherwise = 0	0.544	0.498

**Fig. 1:**
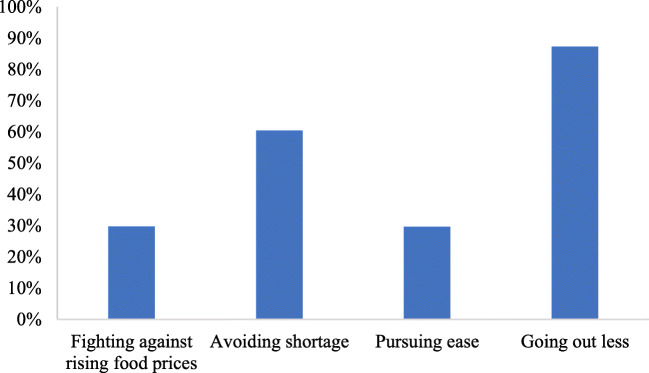
Motivations for food stockpiling

Figure 1 reports the statistics of respondents’ motivations of food stockpiling. Identifying consumer motivations to increase their stockpiling is important to understand their behavior. To do this, respondents were asked what were their motivations for food stockpiling. These motivations include “Going out less,” “avoiding shortage,” “Fighting against rising food prices,” and “pursuing ease,” which cover most of the motivations for food stockpiling. Results in Fig. 1 show that “Going out less” is the main reason for food stockpiling. This is because that most respondents perceive COVID-19 as being highly contagious, and the best prevention is to isolate at home (Ou et al. 2020). Seventy percent of respondents believe COVID-19 is highly contagious. Chinese Government has also restricted travel and encouraged residents across the country to avoid leaving their homes. Sixty percent of respondents stockpile food because they are worried about food shortages. The percentage of respondents who stockpile food because of “Fighting against rising food prices” and “pursuing ease” is 29.71% and 29.63%, separately.

**Fig. 2:**
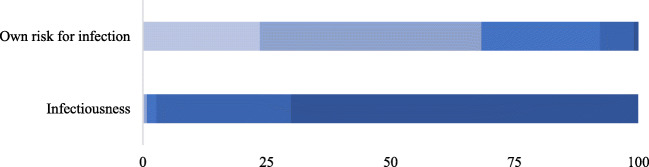
Consumer risk perception of the COVID-19

Two questions (What do you think of the infectivity of COVID-19? How likely do you think you are to be infected with COVID-19?) were included to characterize respondents’ risk perception of COVID-19. Previous studies showed that consumer perception would affect their behavior and preference (Grunert 2005; Gao et al. 2016b). Psychological literature claimed that there were some major psychological pitfalls which often misguide behaviors. “The illusion of control” leads decision-makers to hold exaggerated beliefs about the amount of control they exercise over outcomes. Respondents may perceive the high-risk contagion of COVID-19, but they may systematically overestimate how much control they have. Considering Langer’s (1975) study about the psychological pitfall of “the illusion of control”, this paper compared consumer’s “Perceived infectiousness of COVID-19” (1 = weak infectiousness, 5 = highly contagious) and consumer’s “Perceived own risk for infection of COVID-19” (1 = very low risk, 5 = very high risk). Figure 2 shows that 70.03% of the respondents believed COVID-19 is highly contagious, but only 0.93% of respondents thought they are with very high risk for infection of COVID-19. It implies that there is a psychological pitfall of “the illusion of control” for consumers in COVID-19.

## Results

**Fig. 3:**
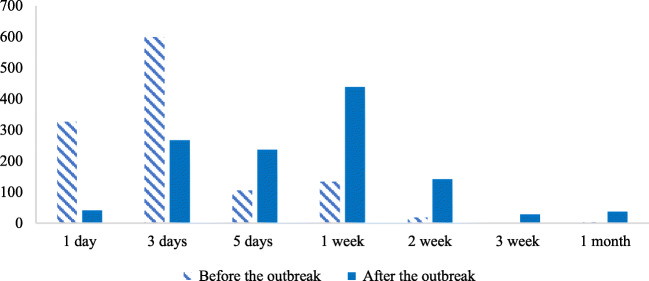
The difference of the scale of food reserves before and after the COVID-19

Figure 3 reports the difference of respondent’s food stockpiling before and after the breakout of COVID-19. The mode of fresh food reserves before COVID-19 was 3.37 days, while the number increased up to 7.37 days after the breakout of COVID-19, showing a significant behavior change. This indicates that the coronavirus pandemic has encouraged many families to expand their fresh products reserves in order to ensure regular levels of consumption, implying a significant demand shock on the food market.

**Fig. 4:**
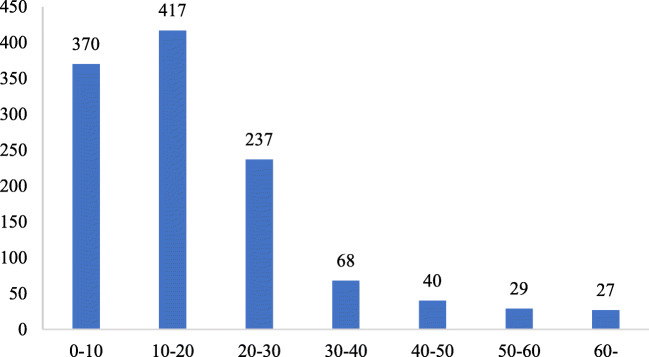
The frequency of WTP for a package of fresh food reserves

Figure 4 reports the statistics of the respondents’ choices. In the survey, it makes sense that most respondents are likely to pay a high premium for fresh food reserves. Note that most respondents were willing to pay a premium of less than 100% for fresh food reserves in COVID-19. The number of respondents decreased when the premium was more than 100%, which is consistent with previous study about consumers’ preferences for green/sustainable food (Yu et al. 2014; Gao et al. 2016b).

We compute the mean values of WTP by assuming the true value is the middle point of the interval, according to the study of Yu et al. (2014). After the breakout of COVID-19, the mean of WTP value for fresh food reserves is 18.14 yuan, implying the increasing value of fresh food reserves for consumers. Although consumers’ WTP values may be over-estimated because they are not actually making a payment (Bohm 1972; Bishop and Heberlein 1990), WTP values estimated by a PC approach are more robust than those relying on a DC approach (Ready et al. 2001). Interestingly, there was a significant drop in the willingness to pay for fresh food reserves as survey time changes, since the data was collected on three consecutive days (20.89 yuan at 11th, 17.59 yuan at 12th, 14.41 yuan at 13th). According to the statistical data of National Health Commission of the People’s Republic of China, the newly confirmed cases of Hubei are 13 cases at 11th, 8 cases at 12th, and 4 cases at 13th, respectively. Especially on the 13th, the number of suspected cases in Wuhan stopped growing for the first time.

The ordered logit model (model 1) was used to analyze factors influencing the scale of food stockpiling, and the interval regression model (model 2) was used to estimate the WTP equation in Stata 14.1. The variables include respondents’ motivation of food stockpiling, consumer risk perception and food stockpiling habit before the outbreak, demographic variables, and survey time. We used the forward, backward, and step-wise selection methods to determine the variables that should be kept in the final models (Table 2).

**Table 2:** Estimation

	Model (1)	Model (2)
Fighting against rising food prices	− 0.196	
	(0.128)	
Avoiding shortage	0.310**	
	(0.124)	
Pursuing ease	0.299**	
	(0.124)	
Going out less	0.360**	
	(0.177)	
Infectiousness	0.235**	− 0.461
	(0.101)	(0.713)
Risk for infection	0.0336	1.190***
	(0.066)	(0.462)
Duration	− 0.0689	0.762**
	(0.043)	(0.310)
Location	− 0.273**	2.465***
	(0.122)	(0.869)
Food stockpiling habit before the outbreak	1.333***	1.576***
	(0.068)	(0.401)
Female	0.289**	0.164
	(0.124)	(0.878)
Age	0.00133	− 1.338
	(0.126)	(0.902)
Education level	0.335**	0.526
	(0.159)	(1.114)
Income2	0.354**	0.354
	(0.147)	(1.041)
Income3	0.435***	3.013***
	(0.158)	(1.119)
Time2	− 0.110	− 2.710***
	(0.140)	(1.007)
Time3	− 0.511***	− 4.936***
	(0.156)	(1.087)

*Significant at 10% level. **Significant at 5% level. **Significant at 1% level

Results of model 1 show that consumers did not stockpile in order to “fight against rising food prices.” This suggests that a stable food supply is more helpful than stable prices to prevent consumer food stockpiling behavior in a crisis like COVID-19. To the extent there may be hard trade-offs between a stable food supply and a stable food price, it shows that policymakers should favor stabilizing food quantity rather than food prices. Food stockpiling behavior is a complex consumer behavior, fueled by a set of multiple motivations and psychological processes. Governments and policymakers should adjust inventory and response strategies in response to the special demand shock in an emergency, like COVID-19 pandemic.

Results of model (1) and model (2) both show that consumers’ risk perception affects their food stockpiling behavior, which is consistent with previous studies about consumer behavior (Gao et al. 2014; Grunert 2005). However, as the above statistics description, there is a psychological pitfall of “the illusion of control” for consumers in COVID-19. Consumer-perceived infectiousness of COVID-19 has significant effects on their real food stockpiling behavior. Consumer-perceived own risk for infection of COVID-19 has significant effect on their willingness to pay for food reserves. It suggests that respondents who feel less in control of COVID-19 were likely to pay more for fresh food reserves in hypothetical question. Interestingly, the effect is weak in their real behavior.

Among demographic variables, most coefficients were significant except age in model (1). Female, high education level, and high-income consumers were more likely to reserve larger scale food reserves. It is consistent with previous studies that female consumers are generally more risk averse than male. However, consumers’ willingness to pay for fresh food reserves is only determined by income in model (2). The coefficients of “Food stockpiling habit before the outbreak” are both significant in the two models. Interestingly, the effect of the survey time is significant, since the survey was conducted in 3 days. Comparing with the samples at the first day, respondents stockpile less scale of food reserve at the third day. This is because consumers could receive timely information about COVID-19 via Internet and TV. They can adjust their food stockpiling strategies based on the timely information.

## Conclusions and discussion

Consumer food stockpiling behavior during COVID-19 pandemic poses a demand shock in the food market. Food stockpiling phenomenon in the coronavirus pandemic in China is similar with the rest of world. Understanding consumer food stockpiling behavior in emergencies can provide important information for governments and policymakers to adjust inventory and response strategies in emergencies. With 1188 valid samples collected from Hubei, Beijing, Shanghai, Guangdong, Zhejiang, Jiangsu, Hebei, and Shanxi during COVID-19 pandemic, this paper analyzed changes of consumer food stockpiling behavior in emergencies.

This paper shows that the coronavirus pandemic encourages many families to stockpile food and expand their fresh products reserves. Respondents stockpiled 3.37 days food reserves before the outbreak of COVID-19, but the number notably increased up to 7.37 days after the outbreak of COVID-19. It implies that food demand would increase dramatically in a short time when there is an emergency, and governments and policymakers should adjust food inventory and response to huge demand shocks during emergencies. The paper also shows that consumers are willing to pay a premium for fresh food reserves during COVID-19 pandemic, and the mean of the WTP premium is 18.14 yuan (60.47%) higher for a package of fresh food reserves. This study of consumer stockpiling behavior in China can help us predict the change of food demand and understand more about consumer preferences in emergencies.

The results from regression analysis further showed that motivations of “Avoiding shortage” rather than “‘fight against rising food prices” have a significant effect on consumer decision about food stockpiling. This result suggests that a stable supply is more helpful than a stable price to reduce consumer food stockpile behavior during COVID-19 pandemic. Policymakers should not ensure a stable food price at the cost of a stable food supply in emergency. Furthermore, we also find that the motivation of “Pursuing ease” and “Going out less” also affect the scale of food stockpiling, which indicates that the food stockpiling behavior is a complex consumer behavior, fueled by a set of multiple and non-overlapping motivations and psychological processes. These factors should be considered by governments and policymakers to adjust their response strategies during COVID-19 pandemic.

Interestingly, we confirmed that there is a psychological pitfall of “the illusion of control” for consumers in COVID-19. Consistent with previous studies about consumer behavior (Gao et al. 2014; Grunert 2005), consumers’ risk perception affects their food stockpiling behavior. However, consumer-perceived infectiousness of COVID-19 has a significant effect on their real food stockpiling behavior, but consumer-perceived own risk for infection of COVID-19 has significant effect on their willingness to pay for food reserves. It suggests that respondents who feel less in control of COVID-19 were likely to pay more for fresh food reserves in hypothetical question. Unsurprisingly, the effect is weaker in their real behavior.

The concerns about food security and consumer food stockpiling behavior often rise during emergencies, which poses a dramatic demand shock on the food market. When societies take emergency measures to cope with issues such as natural disasters and accidents, disasters, public health incidents, and social security incidents, more information about consumer stockpiling behavior and preferences in emergencies should be provided. As of this survey, there were more than 132,000 people who had been diagnosed with COVID-19 pneumonia, and more than 5000 people have lost their lives, according to the latest real-time statistics of WHO. In addition to China, Japan is also facing great pressure from this epidemic. Europe has now become the epicenter of the pandemic, with more reported cases and deaths than the rest of the world combined, apart from China during this survey (WHO 2020). The story of food stockpiling during the COVID-19 pandemic in China is similar with the rest of world. National researchers should therefore share information and work together to meet the challenge of COVID-19 pandemic.

## Data Availability

All data and models used during the study are available from the corresponding author by request.
